# Validation of a Wearable Photoplethysmography-Based Sensor for Compensatory Reserve Measurement Monitoring in Simulated Human Hemorrhage

**DOI:** 10.3390/s26082513

**Published:** 2026-04-18

**Authors:** Jose M. Gonzalez, Ryan Ortiz, Krysta-Lynn Amezcua, Carlos Bedolla, Sofia I. Hernandez Torres, Erik K. Weitzel, Vijay S. Gorantla, Weihua Li, Alexander J. Aranyosi, John A. Rogers, Roozbeh Ghaffari, Victor A. Convertino, Eric J. Snider

**Affiliations:** 1 United States Army Institute of Surgical Research, JBSA Fort Sam Houston, San Antonio, TX 78234, USA; 2 Department of Biomedical Engineering and Chemical Engineering, University of Texas at San Antonio, San Antonio, TX 78249, USA; 3 Graduate School of Biomedical Sciences, University of Texas Health Science Center at San Antonio, San Antonio, TX 78229, USA; 4 United States Air Force 59th Medical Wing, San Antonio, TX 78249, USA; 5 Advocate Health, Wake Forest School of Medicine, Winston-Salem, NC 27101, USA; 6 Epicore Biosystems, Inc., Cambridge, MA 02139, USArooz@epicorebiosystems.com (R.G.); 7 Querrey Simpson Institute for Bioelectronics, Northwestern University, Evanston, IL 60208, USA; 8 Department of Biomedical Engineering, Northwestern University, Evanston, IL 60208, USA; 9 Department of Medicine, Uniformed Services University of the Health Sciences, Bethesda, MD 20814, USA; 10 Department of Emergency Medicine, University of Texas Health Science Center at San Antonio, San Antonio, TX 78229, USA; 11 Department of Surgery, University of Texas Health Science Center at San Antonio, San Antonio, TX 78229, USA

**Keywords:** sensors, hemorrhage, machine learning, wearable healthcare devices, triage, medical devices, signal processing

## Abstract

**Highlights:**

**What are the main findings?**

- The Epicore Epidermal Patch for Imperceptible Care, placed at the triceps, achieved a heart-rate accuracy comparable to a clinical-grade patient finger oximeter when the appropriate filtering techniques were applied.
- Sensor placement critically determines performance, as the Epicore Epidermal Patch for Imperceptible Care demonstrated proper compensatory reserve-tracking trends when placed at the triceps, but not at the clavicle placement site.

**What are the implications of the main findings?**

- Soft, flexible wearable biosensors can enable continuous hemodynamic monitoring in operational environments where traditional finger-based pulse oximetry is impractical.
- Appropriate signal processing can overcome the limitations of non-traditional photoplethysmography measurement sites for hemorrhage detection applications.

**Abstract:**

Hemorrhagic shock remains a leading cause of preventable death in trauma, yet traditional vital signs may fail to reflect early blood loss before physiological compensatory mechanisms are no longer able to maintain hemodynamic stability. The Compensatory Reserve Measurement (CRM) algorithm offers early detection capability using physiological waveforms but requires testing with emerging wearable sensor technologies for operational deployment. This study tested the Epicore Epidermal Patch for Imperceptible Care (EPIC) wearable healthcare device (WHD) for CRM-based hemodynamic monitoring during progressive central hypovolemia induced by lower-body negative pressure (LBNP) to simulate hemorrhage. Twenty participants underwent progressive LBNP while photoplethysmography (PPG) signals were recorded from EPIC sensors placed at the clavicle and triceps alongside a clinical-grade finger pulse oximeter for reference. Signal quality, heart-rate accuracy, and CRM predictions were evaluated across multiple filtering approaches. The triceps placement achieved signal quality comparable to the pulse oximeter reference when Chebyshev Type II filtering was applied, as well as high heart-rate accuracy. CRM derived from the EPIC sensor placed at the triceps tracked compensatory trends during progressive hypovolemia, but prediction magnitudes were inaccurate compared to calculated CRM values. In contrast, the clavicle placement consistently performed poorly across all measurements, regardless of the signal-processing approach. These findings support the feasibility of soft, flexible wearable sensors for continuous hemorrhage monitoring at the triceps location in operational environments where traditional finger-based pulse oximetry is impractical.

## 1. Introduction

### 1.1. Clinical Need for Continuous Hemorrhage Monitoring

Hemorrhagic shock is one of the leading causes of potentially preventable death in both civilian and military prehospital settings [1,2]. In combat casualty care, hemorrhage accounts for approximately 90% of potentially survivable deaths, while civilian trauma indicates that 64% of trauma mortality is attributable to uncontrolled bleeding [3]. The time-critical nature of hemorrhage management is well established—survival probability decreases substantially with each minute of delayed intervention [4].

Treatment involves hemorrhage control followed by fluid resuscitation, preferably using low-titer O whole blood [5]. However, treatment efficacy is expected to become increasingly challenged in future conflicts characterized by limited air and ground evacuation capabilities [6], prolonged field care scenarios [7], and mass casualty events [8]. Civilian prehospital care faces analogous obstacles, including extended transport times in rural and remote regions [9], variable access to prehospital blood products [10], and mass casualty incidents such as mass shootings and natural disasters that strain emergency medical services [11,12]. In both contexts, early and accurate identification of hemorrhage before physiological decompensation is essential to optimize clinical decisions.

A fundamental challenge in hemorrhage detection is the body’s compensatory response to blood loss [13]. Traditional vital signs, including heart rate and blood pressure, can remain within normal ranges until 15–30% of circulating blood volume is lost, at which point compensatory mechanisms begin to fail [14]. Hemorrhagic shock is usually diagnosed and managed in the pre-hospital setting by using subjective indicators and symptoms such as cold and clammy skin, pallor, weak pulse, and vital-sign instability when monitoring is available. These can be imprecise and inconsistent across individuals due to different physiological responses [15]. Critically, these symptoms represent the onset of hemodynamic decompensation rather than early hemorrhage, a stage at which intervention is less effective or potentially too late [16]. This diagnostic gap underscores the need for objective, continuous monitoring technologies capable of detecting the early physiological signatures of hemorrhage before overt decompensation occurs. Importantly, technologies capable of continuously quantifying compensatory reserve could transform triage and monitoring paradigms in both military and civilian trauma systems. Real-time assessment of physiologic reserve may allow for earlier identification of patients at risk for hemodynamic collapse, enabling prioritization of hemorrhage control, blood product resuscitation, or evacuation decisions prior to the deterioration of conventional vital signs. An approach developed to address this diagnostic gap is the Compensatory Reserve Measurement (CRM), which aims to quantify an individual’s remaining physiological tolerance to blood loss.

### 1.2. Lower-Body Negative Pressure

Evaluating hemorrhage-monitoring technologies in human subjects presents ethical and practical constraints, as controlled blood loss studies are limited in their occurrence. Lower-body negative pressure (LBNP) is an experimental procedure that induces central hypovolemia [17]. Volunteers for the procedure are positioned supine with their lower body inside the LBNP chamber, sealed at the waist. Application of progressive negative pressure induces central hypovolemia that mimics the physiological responses observed during actual hemorrhage by causing redistribution of blood volume into the lower half of the body [18].

The hemodynamic responses elicited by LBNP, such as decreased stroke volume, parallels systematic cardiovascular changes observed during progressive hemorrhage [19]. Graded LBNP protocols with stepwise increases in negative pressure produce responses that simulate mild to severe central hypovolemia, with progressive pressure levels being capable of inducing hemodynamic decompensation in individuals undergoing the experimental protocol [20].

LBNP has been extensively used for the development and validation of the CRM algorithm, providing a controlled and reproducible stimulus for characterizing physiological changes associated with progressive hypovolemia [21]. Importantly, the LBNP framework provides the basis for defining the CRM ground truth, as the relationship between applied negative pressure and central blood loss volume enables a quantitative reference standard against which AI model predictions can be evaluated [22]. This model enables standardized comparison of sensor performance across subjects while ensuring participant safety through immediate reversibility upon chamber pressure release [23].

### 1.3. Compensatory Reserve Measurement

The CRM is a metric developed at the U.S. Army Institute of Surgical Research for early triage decision support. CRM estimates the level of physiological compensation an individual has available during hypovolemia, with 100% reflecting full compensation capacity and 0% representing the onset of hemodynamic instability [24]. The ground truth compensatory reserve (*CRM_GT_*), in LBNP experimental protocols, is defined in Equation (1):

(1) $${CRM}_{GT}=1-\frac{LBNP\left(t\right)}{{LBNP}_{HDD}}$$

where *LBNP*(*t*) is the applied negative pressure at time t and *LBNP_HDD_* is the pressure level at which the individual reaches hemodynamic decompensation. This formulation leverages the established linear relationship between applied negative pressure and central blood loss volume, making LBNP the gold-standard experimental model for CRM development and validation [22]. The CRM artificial intelligence (AI) model was developed by leveraging deep learning architectures based on a convolutional neural network (CNN) that is able to automatically extract changing features from high-fidelity waveforms [25]. CRM aims to allow clinicians to assess and treat patients in a timely manner who otherwise show no signs of hemodynamic decompensation based on traditional vital signs. While CRM has been validated using high-fidelity clinical-grade volumetric-clamp arterial waveform signals, the clinical utility of CRM ultimately depends on the sensing hardware used to acquire the underlying physiological signals.

### 1.4. Epicore Sensor System

The Epidermal Patch for Imperceptible Care (EPIC) system (Epicore Biosystems, Inc., Cambridge, MA, USA) is a wearable healthcare device (WHD) designed to detect quantitative physiologic metrics, including photoplethysmography (PPG) signals and motion-related vital signs [26,27,28]. The EPIC system represents an emerging class of soft, flexible electronic systems that enable complete non-invasive sensing of pulse waveforms necessary for CRM [29]. Unlike traditional clinical monitoring devices that require rigid sensors and controlled environments, the EPIC system is comparable in size and geometry to a soft gauze bandage, allowing for unobtrusive placement on various anatomical locations. The system supports continuous monitoring for up to 24 h and wirelessly transmits data to a mobile device for data storage and real-time analysis.

While sensor utility in sweat biomarker analysis and hydration monitoring has been demonstrated by Epicore Biosystems [27,30], the EPIC WHD evaluated in this study represents the first iteration of the platform configured for hemodynamic sensing under hypovolemic conditions. Unlike prior configurations designed for sweat biomarkers or hydration analyses, this system was optimized for PPG acquisition during changing physiological conditions induced by the LBNP. Furthermore, wearable PPG waveforms may differ morphologically from the high-fidelity signals on which CRM was originally developed, as factors including motion artifacts, tissue optical properties, and sensor-skin coupling can influence waveform characteristics. Validation of the EPIC system PPG sensors for hemodynamic monitoring requires systematic assessment across signal quality, accuracy of derived physiological parameters, and performance of the CRM algorithm under conditions of progressive hypovolemia. Establishing the EPIC system’s suitability for CRM-enabled hemodynamic monitoring necessitates rigorous evaluation in controlled hypovolemic conditions.

### 1.5. Study Objective and Hypothesis

The objective of this study was to validate the EPIC WHD for hemodynamic monitoring using the CRM algorithm during simulated hemorrhage using LBNP. Two anatomical sensor placements (clavicle and triceps) were evaluated alongside a reference clinical-grade finger pulse oximeter. Specifically, we evaluated (1) PPG signal-quality metrics, including signal-to-noise ratio and signal-quality index across multiple filtering approaches; (2) heart-rate measurement accuracy compared to electrocardiogram (ECG)-derived ground truth; and (3) CRM algorithm predictions. We hypothesized that a wearable PPG sensor placed at a vascularized peripheral location would produce PPG signals of adequate quality for demonstrating physiological tracking trends comparable to a clinical-grade pulse oximeter reference as well as possessing sufficient quality for CRM estimation.

## 2. Materials and Methods

This study employed a structured validation framework to evaluate the feasibility of WHD PPG sensors for hemorrhage prediction via CRM. In addition, the signals underwent validation to ensure that the wearable sensors produced physiologically accurate and high-quality signals. The analysis contained three distinct stages: (1) signal-quality characterization to quantify signal-to-noise and signal-quality index metrics to determine whether the wearable PPG waveform retains sufficient fidelity for downstream processing; (2) heart-rate estimation accuracy to serve as a benchmark of sensor performance against clinical-grade references; and (3) CRM algorithm predictions to assess whether WHD PPG waveforms can support compensatory status prediction. This stepwise approach ensures that any observed limitations in CRM performance can be attributed to algorithmic factors rather than underlying signal-quality deficiencies. As the WHD PPG sensor employed in this study is not a commercially validated medical device, a clinical pulse oximeter was also evaluated using the same approach to provide a clinical reference.

### 2.1. Epicore Sensor Specifications

**Figure 1:**
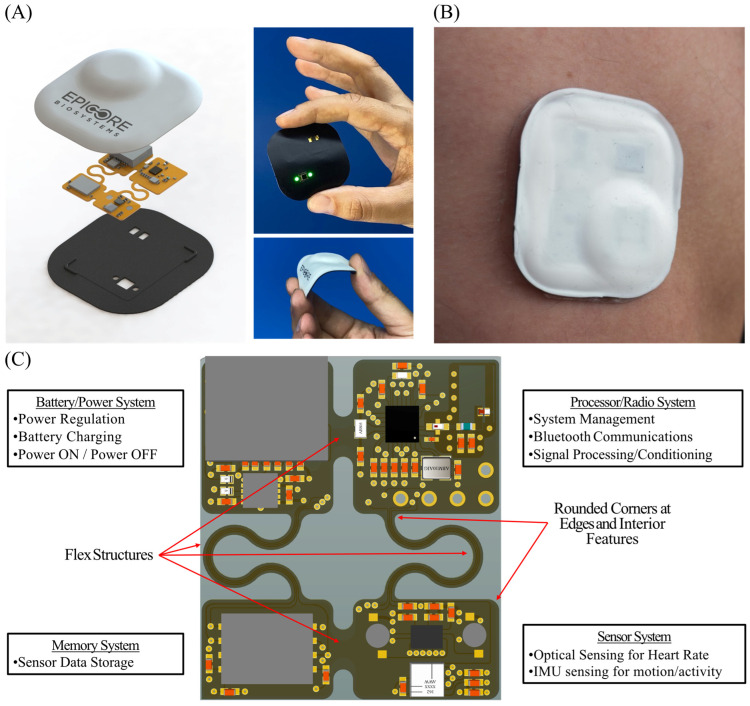
EPIC WHD schematic drawings and photographs. (**A**) Exploded view of the EPIC WHD, showing the circuit board and flexible silicone encapsulation. The black surface faces the skin and has openings for the PPG optical sensor and for connecting to an external charger (Left). Photographs of the EPIC WHD showing the PPG sensor and charger opening (Right). (**B**) Photograph of the EPIC WHD worn on the triceps with a skin-interface bioadhesive layer. (**C**) Expanded view of circuit layout, highlighting critical components such as the battery, IMU, PPG, and microcontroller. The flex connectors allow the circuit to conform to the shape of the human body.

The EPIC system comprises three main systems: a microcontroller/communications system, a sensor system, and a power management system. Each system is modular to support future hardware updates. The device has an onboard microprocessor and radio for algorithmic processing and wireless data transfer to a compatible smartphone application. The sensor system is housed in soft polymeric sheets containing highly flexible electronics coupled to a thin-film substrate (Figure 1A).

The microcontroller/communications system handles primary communications using Bluetooth 5 wireless protocol and operates the flexible hybrid electronics (FHE) prototype EPIC WHD. It interacts with the sensor system, retrieving data and transmitting it to a compatible smart device. The main processor for the EPIC system was the Nordic nRF52832 (Nordic Semiconductor, Trondheim, Norway), as it is compatible with modern data-processing algorithms and power optimization techniques (Figure 1C).

The sensor system contains the motion and optical sensors onboard the FHE module. The former is an inertial measurement unit (IMU) that provides context about user movements and activities, while the latter relies on light-emitting diodes (LEDs) and photodetectors to create PPG signals. The optical front-end utilizes an Analog Devices MAX30110 (Analog Devices, Wilmington, MA, USA) analog front-end in a reflectance-mode configuration with two green LEDs and one photodetector at a center-to-center separation distance of 4.25 mm. A green LED was selected for use in the EPIC system’s PPG, as it offers a balance of signal quality in the presence of motion artifacts and power efficiency for use in PPG sensors, ideal for cardiovascular monitoring across anatomical regions of the body [31]. For motion sensing, the EPIC system used a TDK ICM-20602 (TDK Corporation, Chuo City, Tokyo, Japan) IMU in this module, which is a 6-axis microelectromechanical system sensor that has a tri-axis accelerometer and a gyroscope with a sampling rate in the kHz range (Figure 1C).

The power management system uses a 3.8 V lithium battery cell as the input and outputs two system voltages via the LP5996 (Texas Instruments, Dallas, TX, USA) low-dropout regulator. The system voltages are +1.8 V and +3.3 V and feed the remainder of the systems for optimal power and efficiency. The +3.3 V supply is configured to power the LEDs used in the optical sensor. This is a necessity, as the green LEDs only conduct current at a minimum of +3.0 V (Figure 1C).

Two EPIC WHD locations were evaluated: the right mid-clavicular anterior chest (clavicle) and the right triceps’ posterior surface (triceps) (Figure 1B). For simplicity, when discussing specifics for the EPIC WHDs at these two locations, they are referred to as WHD Clavicle and WHD Triceps throughout the text. PPG data were recorded at a sampling frequency of 100 Hz and transmitted wirelessly to the EPIC mobile application for storage.

### 2.2. Experimental Protocol

Datasets were captured through an Institutional Review Board-approved human research protocol. Potential study participants were familiarized with study requirements and given a chance to review the consent form. The inclusion criteria for enrollment were (1) normotensive (<140/90) males or females, (2) between 18 and 65 years old, (3) who were military or civilians, (4) had a waist circumference between 22 and 42 inches, and (5) were willing to refrain from exercise and stimulants (including caffeine, alcohol, chocolate, and herbal medications) for the 24 h prior to the study. Once enrolled in the study, the participants completed a health questionnaire and screening procedure with an Advanced Cardiovascular Life Support-certified medical monitor who verified compliance to withholding requirements 24 h prior to the study, exclusion criteria, and measurements for wearable sensors to be used during the study. Once the participant satisfactorily passed screening, they were fitted with a kayak-style neoprene skirt appropriate to their waist size and secured to the LBNP chamber.

Once the participant was secured, sensors were placed at appropriate locations. For this work, required sensors included a finger pulse oximeter (Clinical PulseOx; Masimo, Irvine, CA, USA) connected to a Dräger infinity patient monitor (Lübeck, Germany) and a 3-lead ECG connected to a Finapres Nova (Finapres Medical Systems, Enschede, The Netherlands) for clinical monitoring. These sensors were used for data collection in addition to the EPIC WHDs placed at the clavicle and triceps locations. All data streams were temporally synchronized by recording both device time and master clock time at a common rollover event (e.g., HH:MM:00), with timestamp offsets corrected post hoc to align all signals to a common time reference prior to analysis.

Following sensor placement, participants underwent a standardized LBNP protocol. The protocol began with a 5 min baseline period at atmospheric pressure (0 mmHg), followed by progressive stepwise reductions in chamber pressure. Negative pressure was increased in increments of −15 mmHg every 5 min until reaching −60 mmHg, after which pressure was incremented by −10 mmHg until the participant reached presyncope or completed the protocol at −100 mmHg, whichever occurred first. Presyncope was defined as the onset of symptoms, including lightheadedness, nausea, tunnel vision, or a sudden drop in systolic blood pressure below 80 mmHg [32,33]. Upon reaching presyncope or protocol completion, chamber pressure was immediately released to atmospheric pressure and participants were monitored over a 10 min recovery period.

### 2.3. Data Processing Pipeline

#### 2.3.1. Signal-to-Noise Ratio Analysis

High-fidelity Clinical PulseOx waveform data was resampled to 100 Hz for this analysis to mimic the CRM AI model input size as well as the default recording frequency of the Epicore WHDs. Resampling was performed by decimating the waveform data, originally sampled at 1 kHz, by selecting every 10th sample. Signal quality was assessed by calculating signal-to-noise ratio (SNR) for each PPG waveform (Clavicle, Triceps, Clinical PulseOx).

A rolling-window approach was used to compute sample-wise SNR estimates with a 5 s window and a stride of 1 s. For each 5 s window (500 samples), a fourth-order Butterworth high-pass filter was applied to remove the DC offset and baseline drift to isolate the pulsatile (AC) component of the PPG signal. The AC-coupled signal was decomposed into signal and noise components using two fourth-order Butterworth filters with a 5 Hz cutoff, selected as the frequency that has been shown to encompass the pulsatile component of the PPG signal [34]. This cutoff fully captures the fundamental cardiac frequency across the heart range observed in this study. The low-pass filtered output (≤5 Hz) captured the cardiac pulsatile component, while the high-pass filtered output (>5 Hz) represented high-frequency noise. *SNR* was then calculated, in decibels, using Equation (2):

(2) $$SNR\left(dB\right)=20\times \log_{10}\frac{{RMS}_{Signal}}{{RMS}_{Noise}}$$

where *RMS* represents the root mean square of the low-pass (signal) and high-pass (noise) filtered components, respectively. The window was systematically moved forward 1 s throughout the entire signal duration. The median SNR across all windows was then calculated for each respective PPG waveform per subject.

#### 2.3.2. Signal-Quality Index Analysis

For the signal-quality index (SQI) analysis, the Clinical PulseOx waveform data was processed identically to the SNR analysis and resampled to 100 Hz to match the CRM AI model input requirements and the native EPIC WHD sampling frequency. This enabled direct comparison across sensor placements and filtering strategies. Representative raw PPG waveforms from each device before signal processing are shown in Supplementary Figure S1. Signal processing was implemented to isolate physiologically relevant pulsatile components of the PPG waveform while minimizing motion artifacts and baseline drift inherent to wearable sensor acquisition. SQI was assessed using both Masimo and raw Epicore WHD signals, in addition to signals filtered with a Butterworth bandpass filter (SciPy v1.16), a Chebyshev Type II bandpass filter (SciPy v1.16), and a customized filter (MATLAB 2018b).

The Butterworth bandpass filter was implemented as a fourth-order infinite impulse response (IIR) filter with a passband of 0.5–8.0 Hz, selected to encompass the fundamental cardiac frequency and its harmonics while attenuating baseline drift and high-frequency noise [35]. The Chebyshev Type II bandpass filter was similarly implemented as a fourth-order IIR filter with a passband of 0.5–8.0 Hz and a stopband attenuation of 40 dB. This filter design provides a maximally flat passband while concentrating ripple in the stopband, offering sharper roll-off characteristics compared to the Butterworth design [36]. IIR filters were applied using zero-phase forward–backward filtering to eliminate phase distortion.

The Custom filter is a linear-phase finite-impulse response (FIR) filter designed in MATLAB (MathWorks, Natick, MA, USA). The filter was applied via one-dimensional convolution with a fixed 500-sample delay to align outputs with corresponding inputs. Padding at signal boundaries was performed using constant edge replication to minimize edge artifacts. The filter coefficients were supplied directly by Epicore Biosystems. The MATLAB implementation was converted to Python v3.12 for integration with the analysis pipeline.

Signal-quality assessment was performed on all three PPG waveforms (WHD Clavicle, WHD Triceps, Clinical PulseOx) using a sliding-window approach with a window size of 5 s and a stride of 1 s. PPG peaks were detected using a peak detection algorithm based on Elgendi et al. [37]. Beat-to-beat analysis windows surrounding each peak were then input into Neurokit2’s PPG quality function alongside the peak themselves, using the template-matching methodology based on Orphanidou et al. [38,39]. Briefly, this approach quantified beat-to-beat morphological consistency by computing the mean Pearson correlation coefficient between individual pulse waveforms and a window-averaged reference template, yielding a continuous quality score between 0 and 1. The window was systematically moved forward 1 s throughout the entire signal duration. The median SQI across all windows was then calculated for each respective PPG waveform per subject. In cases where SQI could not be computed due to signal morphology (e.g., excessive baseline wander or absence of detectable peaks), SQI was assigned a value of 0.

#### 2.3.3. Heart-Rate Correlation Analysis

For each subject dataset, heart-rate time series were derived from 100 Hz raw and filtered PPG signals (Butterworth filter, Chebyshev Type II filter, Custom filter) for both EPIC WHD locations (clavicle, triceps) and the Clinical PulseOx using LabChart 8 Pro (ADInstruments, Sydney, Australia). Ground-truth heart rate was derived from the ECG signal using the same software. Prior to correlation analysis, heart-rate values of the gold-standard ECG heart rate were screened for clinical plausibility, with values outside the range of 30–200 bpm excluded as artifacts [40,41].

### 2.4. CRM AI Model

The CRM quantifies the percentage of remaining capacity to compensate in a human subject experiencing central hypovolemia, with a value of 100% indicating full ability to compensate and 0% indicating impending cardiovascular decompensation [22]. The pre-trained CNN model used in this study was developed and validated by Roden et al. [42] using volume-clamp-derived arterial blood pressure waveforms and clinical pulse oximeter PPG. Here, it is applied without modification to evaluate its performance with wearable PPG inputs [43].

The algorithm requires a 100 Hz PPG waveform; thus, high-fidelity waveforms from a clinical-grade pulse oximeter were resampled using decimation by selecting every 10th value, as the original sampling was 1 kHz. The EPIC WHD was natively recorded at a frequency of 100 Hz, so no down-sampling was necessary. The CNN model requires a 5 s window, so inputs were 500 samples at 100 Hz. In addition, the waveform must be normalized to a 0 to 1 scale using a min–max scaler:

(3) $$X^{\prime }=\frac{x_{i}-\min\left(x\right)}{\max\left(x\right)-\min\left(x\right)}$$

where *x_i_* represents each sample point in the PPG waveform time window, and the minimum and maximum amplitudes are represented by the min(*x*) and max(*x*), respectively.

The CNN AI model generated CRM predictions at 1 Hz, computed using a 5 s sliding window with a 1 s stride. To reduce variability in the CRM output, a 20 s centered moving average was applied to the raw predictions. This smoothing was implemented using a rolling mean (window = 20 samples, centered = True), where each smoothed value incorporates 10 s of preceding and 10 s of subsequent CRM estimates. A centered window was selected for this offline analysis to minimize phase distortion in the smoothed signal [44].

### 2.5. Performance Metrics

The performance of heart-rate estimation and the CRM model predictions was evaluated using multiple complementary metrics to characterize accuracy, bias, and clinical utility. Coefficient of determination (R^2^) was used to assess the measure of overall agreement between sensor outputs compared to the reference ground truth (CRM_GT_). Median error (MdE) was used to characterize systematic bias in heart-rate estimation and CRM predictions, while median absolute error (MdAE) quantified overall accuracy independent of bias direction. Accuracy, as defined by ANSI/AAMI EC13 standards [45], was calculated as the percentage of measurements within ±10% or ±5 bpm of the reference ECG heart rate, whichever threshold is greater. Trend detection time was used to evaluate the clinical utility of early hemorrhage warning by observing the slope trends of CRM predictions, where a significant trend was set as a slope drop of 15% CRM over a sliding 5 min window.

### 2.6. Statistical Analysis

For statistical comparisons, we first assessed normality using the Shapiro–Wilk test, where *p* < 0.05 indicated non-normal distribution. As normality assumptions were not met for comparisons, a non-parametric Kruskal–Wallis test and post hoc Dunn’s multiple-comparison test were used throughout. Analyses were performed for pairwise comparisons for SNR and SQI results, heart-rate correlation results, CRM prediction performance results, and trend detection results. Statistical tests are described in figure captions when applicable. Throughout, *p*-values < 0.05 indicated statistical significance. Statistical significance is denoted on figures for ease of interpretation, and precise *p*-values are provided in the accompanying text.

## 3. Results

### 3.1. Study Population

Through an IRB-approved protocol, datasets were collected from 20 participants who completed the study protocol, of the 23 recruited in total. Of the three participants for whom datasets were not collected, two did not pass study screening prior to the experimental procedure and the other participant chose not to participate. The participant population was recruited from the general San Antonio, TX, USA area. Demographic data are summarized in Table 1. All 20 participants had data recorded from the clinical sensors and the experimental WHD Triceps location throughout the duration of the experiment. Issues related to connectivity resulted in only 17 participant datasets being captured for the WHD Clavicle. On average, participants made it to LBNP step −70 mmHg, and most experiments were stopped by the medical monitor.

### 3.2. Signal-to-Noise Ratio

**Figure 2:**
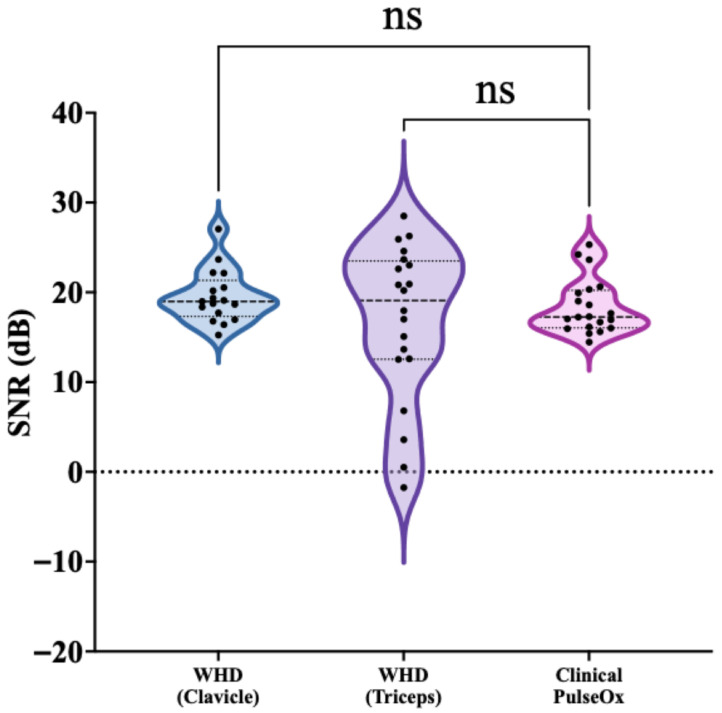
SNR across all subjects per WHD (Clavicle and Triceps) and Clinical PulseOx. Violin plots show the distribution of SNR values with each group; black dots indicate individual subject measurements, and the internal horizontal lines denote the median and interquartile range. Statistical significance was determined by a Kruskal–Wallis test and post hoc Dunn’s multiple-comparison test to compare the three groups (ns = not significant or *p*-value > 0.05).

SNR was calculated to assess the quality of PPG signals acquired from each sensor placement (Figure 2). The WHD Clavicle demonstrated the most consistent SNR of 19.53 ± 2.93 dB across 17 participants and had a range of 15.25 to 27.05 dB. The WHD Triceps exhibited a mean SNR of 16.72 ± 8.79 dB across 20 participants and demonstrated variability across subjects, with values ranging from −1.75 to 28.50 dB. The Clinical PulseOx had an SNR of 18.40 ± 3.08 dB across 20 participants, with a more constrained range of 14.45 to 25.31 dB compared to the triceps placement. Statistical comparison revealed no significant difference in SNR between the WHD Clavicle and the Clinical PulseOx reference (*p* = 0.5755). The WHD Triceps placement also demonstrated no significant difference in SNR compared to the Clinical PulseOx (*p* > 0.9999).

### 3.3. Signal Quality Index

#### 3.3.1. Raw Signal

**Figure 3:**
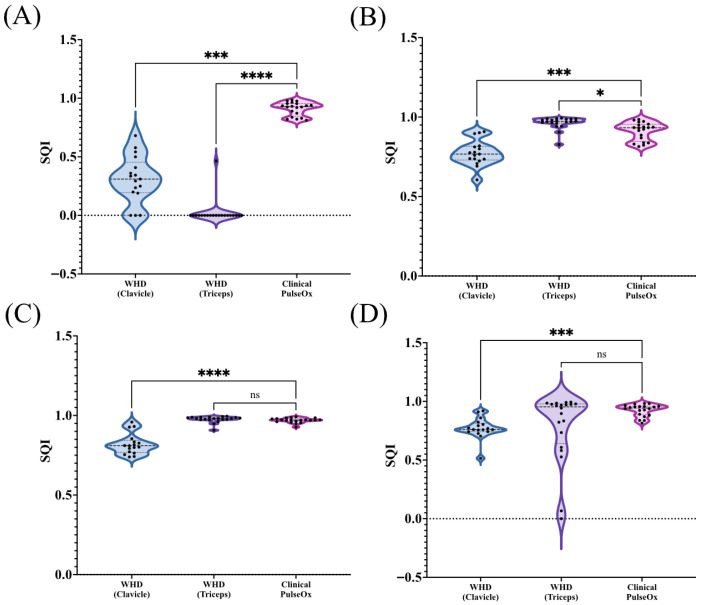
SQI across all subjects per WHD (Clavicle and Triceps) and Clinical PulseOx for the (**A**) raw PPG signal; (**B**) Butterworth-filtered PPG signal; (**C**) Chebyshev Type II-filtered PPG signal; (**D**) Custom-filtered PPG signal. Violin plots show the distribution of SQI values with each group; black dots indicate individual subject measurements, and the internal horizontal lines denote the median and interquartile range. Statistical significance was determined by a Kruskal–Wallis test and a post hoc Dunn’s multiple-comparison test to compare the three groups (ns = non-significant, *p* > 0.05; * *p* ≤ 0.05; *** *p* ≤ 0.001; **** *p* ≤ 0.0001).

SQI was computed to provide an assessment of PPG waveform quality based on waveform morphology and reliability across sensor placements (Figure 3A). The Clinical PulseOx demonstrated the highest SQI with a mean value of 0.91 ± 0.06 across 20 participants, with values ranging from 0.81 to 0.99. The WHD Triceps achieved an SQI of 0.02 ± 0.10 across 20 participants, with only one participant successfully having an SQI calculated. The WHD Triceps had a variability spanning 0 to 0.47. The WHD Clavicle had a mean SQI of 0.31 ± 0.20 across 17 subjects and had a range of SQI values between 0 and 0.68. Statistical analysis revealed significant differences between sensor placements. The Clinical PulseOx demonstrated significantly higher SQI compared to the WHD Triceps (*p* < 0.0001) and the WHD Clavicle (*p* = 0.0002).

#### 3.3.2. Butterworth-Filtered Signal

Using a Butterworth filter, the WHD Triceps demonstrated a dramatic improvement in SQI, with the highest mean value of 0.96 ± 0.038 and overall values ranging from 0.83 to 0.99 (Figure 3B). The Clinical PulseOx followed with a mean SQI value of 0.91 ± 0.06 and values ranging between 0.82 and 0.99 among the same 20 participants. Finally, the WHD Clavicle followed, with the lowest mean SQI of 0.77 ± 0.08 and SQI values spanning 0.61 to 0.91 across a total of 17 participants. Statistical analysis revealed significant differences between sensor placements and the Clinical PulseOx. The WHD Triceps sensor demonstrated significantly higher SQI compared to the clinical reference (*p* = 0.0171), while the WHD Clavicle had significantly lower SQI than the clinical reference (*p* = 0.0008).

#### 3.3.3. Chebyshev Type II-Filtered Signal

Using the Chebyshev Type II filter, the WHD Triceps demonstrated the highest SQI with a mean value of 0.98 ± 0.02 across 20 participants, with values ranging from 0.91 to 1.00 (Figure 3C). The Clinical PulseOx achieved a comparable mean SQI of 0.97 ± 0.02 across 20 participants, with values ranging from 0.93 to 0.99. The WHD Clavicle exhibited the lowest mean SQI of 0.82 ± 0.07 across 17 participants, with values spanning 0.74 to 0.96. Statistical analysis revealed no significant difference in SQI between the WHD Triceps and the clinical reference (*p* = 0.176). However, the WHD Clavicle demonstrated significantly lower SQI compared to the clinical reference (*p* < 0.0001).

#### 3.3.4. Custom-Filtered Signal

Using the Custom filter, the Clinical PulseOx demonstrated the highest SQI with a mean value of 0.93 ± 0.05 across 20 participants, with values ranging from 0.82 to 0.98 (Figure 3D). The WHD Triceps achieved a mean SQI of 0.79 ± 0.30 across 20 participants; however, this placement exhibited substantial variability with values ranging from 0 to 0.99. The WHD Clavicle demonstrated a mean SQI of 0.77 ± 0.09 across 17 participants, with values spanning 0.52 to 0.92. Statistical analysis revealed no significant difference in SQI between the WHD Triceps and the clinical reference (*p* = 0.793), despite the high variability observed in the triceps location. The WHD Clavicle demonstrated significantly lower SQI compared to the clinical reference (*p* = 0.0003).

### 3.4. Heart-Rate Analysis

**Figure 4:**
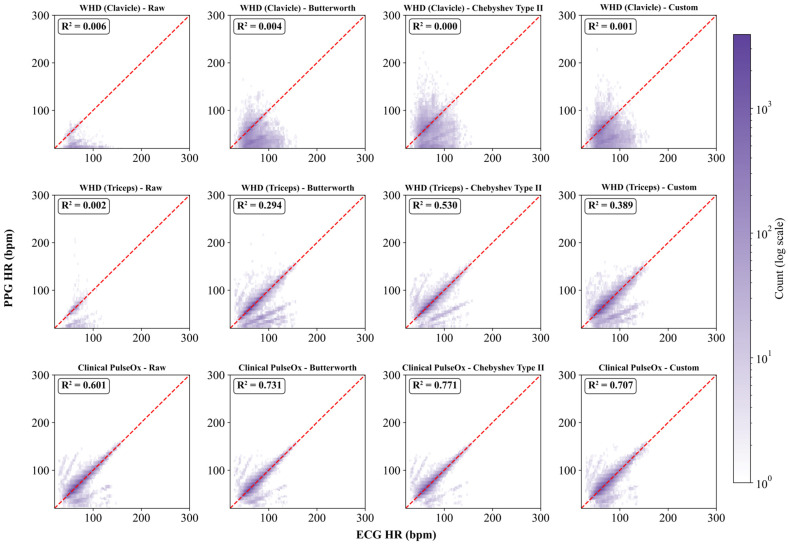
Density scatterplots of PPG-derived heart rate versus ECG-derived heart rate across sensor placement and filter conditions. The dashed red line indicates the identity line, visually representing perfect correlation. Color intensity represents point density on a logarithmic scale. The coefficient of determination is displayed for each condition.

Correlations were computed between heart-rate time-history data derived from the Clinical PulseOx and the EPIC WHD (Clavicle and Triceps) PPG waveforms to evaluate physiological signal quality and reliability relative to the ECG ground-truth reference (Figure 4).

**Figure 5:**
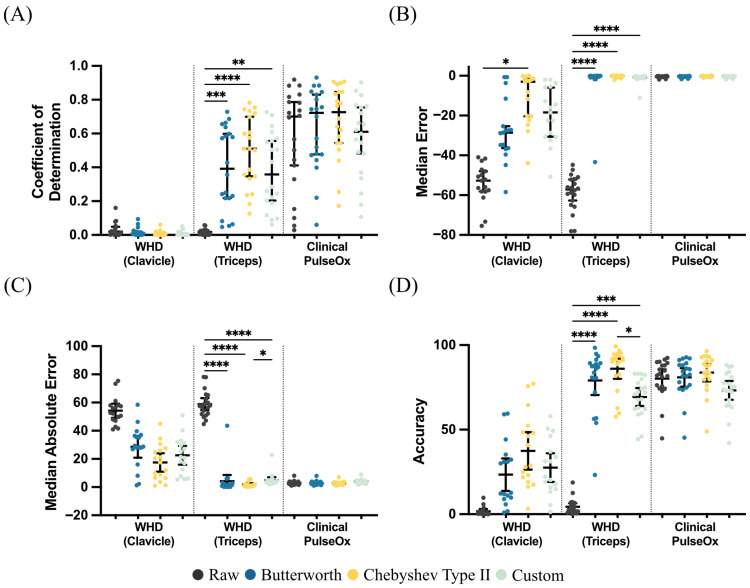
Performance metrics related to heart-rate prediction for (**A**) coefficient of determination, (**B**) median error, (**C**) median absolute error, and (**D**) accuracy. Statistical significance was determined by a Kruskal–Wallis test and a post hoc Dunn’s multiple-comparison test to compare filtering approaches for each sensor placement or type (* *p* ≤ 0.05; ** *p* ≤ 0.01; *** *p* ≤ 0.001; **** *p* ≤ 0.0001).

Correlations between the PPG-derived heart rates and ECG-derived heart rates were assessed. Heart rate derived from the Clinical PulseOx showed strong agreement with heart rate derived from the ECG signal, with R^2^ values of 0.601, 0.731, 0.771, and 0.707 for raw, Butterworth-filtered, Chebyshev Type II-filtered, and Custom-filtered data, respectively (Figure 5A). The Chebyshev Type II filter yielded the highest agreement. Heart rate derived from the WHD Triceps showed moderate agreement when filtered using the Chebyshev Type II filter (R^2^ = 0.530). Agreement was weak for the Butterworth and Custom-filtered data (R^2^ = 0.294 and 0.389, respectively), while the raw signal showed poor agreement (R^2^ = 0.002). Heart rate derived from the WHD Clavicle showed negligible agreement across all filtered conditions (R^2^ ≤ 0.006), indicating that heart rate could not be reliably extracted from this sensor location.

MdE was assessed between the PPG-derived heart rates and the ECG-derived heart rates (Figure 5B). For the Clinical PulseOx, MdE was −0.37, −0.36, −0.27, and −0.31 bpm for the raw PPG signal, the Butterworth-filtered signal, the Chebyshev Type II-filtered signal, and the Custom-filtered signal, respectively. For the WHD Triceps, MdE was the highest for the raw signal, with a MdE of −57.11 bpm. After filtering, MdE dropped to −0.24 bpm for the Butterworth filter, −0.11 bpm for the Chebyshev Type II filter, and −0.64 bpm for the Custom filter. The WHD Clavicle had high MdEs, with a −52.73 bpm error for the raw signal, −28.54 bpm for the Butterworth filter, −3.02 bpm for the Chebyshev Type II filter, and −18.46 bpm for the Custom filter.

MdAE was also assessed between the PPG-derived heart rates and the ECG-derived heart rates (Figure 5C). For the Clinical PulseOx, MdAE was 2.74, 2.70, 2.54, and 3.80 bpm for the raw signal, the Butterworth-filtered signal, the Chebyshev Type II-filtered signal, and the Custom-filtered signal. For the WHD Triceps, MdAE for the raw, Butterworth-filtered, Chebyshev Type II-filtered, and Custom-filtered PPG signals was 57.72, 1.46, 1.20, and 4.04 bpm, respectively. For the WHD Clavicle, MdAE was 52.75 bpm for the raw signal, 28.77 bpm for the Butterworth-filtered signal, 17.68 bpm for the Chebyshev Type II-filtered signal, and 21.42 bpm for the Custom-filtered signal.

In addition, the accuracy of heart rate was assessed with all PPG signal inputs and the various filters (Figure 5D). The assigned threshold for accuracy was within ±10% or ±5 bpm of the reference ECG heart rate, per ANSI/AAMI EC13 standards. Accuracy from the Clinical PulseOx showed high median accuracies of 82.46%, 83.45%, 86.02%, and 74.60% for raw, Butterworth-filtered, Chebyshev Type II-filtered, and Custom-filtered data, respectively. The WHD Triceps exhibited high heart-rate accuracies, aside from the raw signal (2.81%), of 86.54%, 91.22%, and 71.46% for the Butterworth-filtered, Chebyshev Type II-filtered, and Custom-filtered PPG signals, respectively. Finally, the WHD Clavicle demonstrated poor heart-rate accuracies across all conditions, with a median accuracy of 0.69%, 12.69%, 28.71%, and 22.09% for the raw, Butterworth, Chebyshev Type II, and Custom-filtered PPG signals, respectively.

Comparison of filter types by different performance metrics for each sensor placement revealed significant differences in heart-rate estimation performance across all sensor-filter combinations (Figure 5). For the coefficient of determination (Figure 5A), no significant differences were observed between filter conditions for the WHD Clavicle or Clinical PulseOx. For the WHD Triceps, the raw signal showed significantly lower R^2^ compared to Butterworth (*p* = 0.0008), Chebyshev Type II (*p* < 0.0001), and Custom (*p* = 0.0012)-filtered signals, with no significant differences among the three filters. For MdE (Figure 5B), the clavicle WHD showed one significant comparison with raw, demonstrating greater bias than Chebyshev Type II (*p* = 0.0338). The triceps WHD showed significantly greater bias for the raw signal compared to all filtered conditions (all *p* < 0.0001), with no significant differences among the filters. No significant differences were observed for the Clinical PulseOx. For MdAE (Figure 5C), no significant differences were observed for the clavicle WHD or Clinical PulseOx. The triceps WHD showed significantly higher error for the raw signal compared to all filtered conditions (all *p* < 0.0001). Additionally, Chebyshev Type II filtering achieved significantly lower MdAE than Custom filtering (*p* = 0.0365). For accuracy (Figure 5D), no significant differences were observed for the clavicle WHD or Clinical PulseOx. The triceps WHD showed significantly lower accuracy for the raw signal compared to the Butterworth (*p* < 0.0001), Chebyshev Type II (*p* < 0.0001), and Custom (*p* = 0.0001)-filtered signals. Chebyshev Type II filtering also achieved significantly higher accuracy than Custom filtering (*p* = 0.0487).

### 3.5. CRM Algorithm Performance

#### 3.5.1. WHD Clavicle

**Figure 6:**
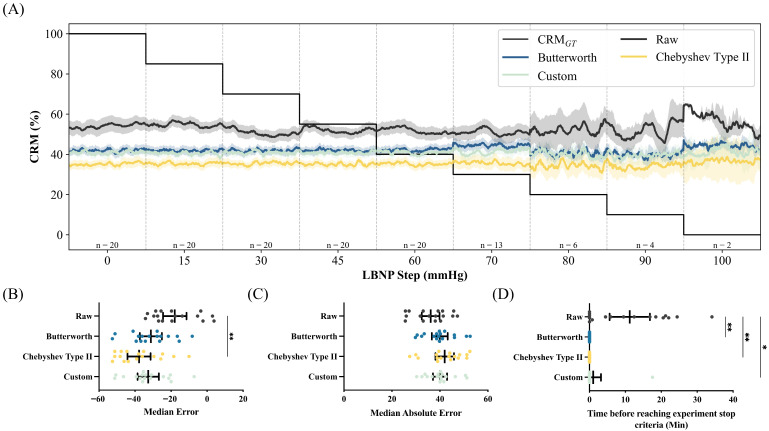
Results for WHD Clavicle sensor-based CRM predictions. (**A**) WHD Clavicle CRM over normalized LBNP steps across various filters compared against CRM_GT_. Performance for (**B**) median error, (**C**) median absolute error, and (**D**) trend time for CRM predictions. Statistical significance was determined by a Kruskal–Wallis test and a post hoc Dunn’s multiple-comparison test to compare performance metrics for each filtering type (* *p* ≤ 0.05; ** *p* ≤ 0.01).

CRM predictions from the WHD Clavicle demonstrated poor agreement with CRM_GT_ across all filtering conditions (Figure 6A). The raw signal yielded highly variable CRM estimates that failed to track the expected decline during the progression of LBNP. The application of Butterworth, Chebyshev Type II, and Custom filters did not improve performance, with all filtered outputs showing no correlation with the ground-truth trajectory.

MdE revealed strong systematic bias across all filter conditions (Figure 6B), with the raw signal showing the least bias at −19.74%, followed by Butterworth (−32.20%), Custom (−34.79%), and Chebyshev Type II (−39.05%). The negative bias indicates consistent underestimation of CRM relative to the ground truth. MdAE exceeded 38.00% for all applied filters (Figure 6C), with values of 38.08% (raw), 39.78% (Butterworth), 40.26% (Custom), and 43.76% (Chebyshev Type II). Statistical analysis demonstrated that the MdE between filtering conditions was significant between the raw and the Chebyshev Type II filter (*p* = 0.0066) but showed no statistical significance with the Butterworth (*p* = 0.1474) and Custom filter (*p* = 0.0691). MdAE was not significant for all filter conditions compared to the raw signal (All *p* > 0.9999).

Trend detection time analysis demonstrated that the clavicle WHD failed to reliably detect threshold crossings, with median detection times of 0 min across all filtered conditions except raw, which had a mean trend detection time of 11.23 min for identifying hemodynamic decompensation (Figure 6D). Raw signal detection times were significantly earlier than Butterworth (*p* = 0.0038), Chebyshev Type II (*p* = 0.0038), and Custom-filtered (*p* = 0.0132) conditions.

#### 3.5.2. WHD Triceps

**Figure 7:**
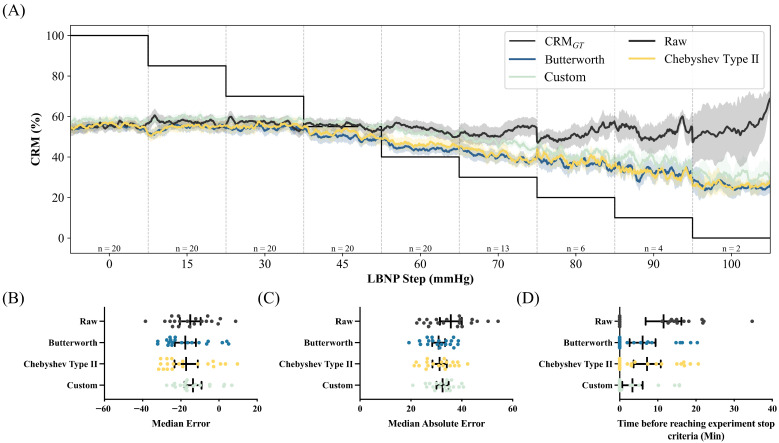
Results for WHD Triceps sensor-based CRM predictions. (**A**) Triceps WHD CRM over normalized LBNP steps across various filters compared against CRM_GT_. Performance for (**B**) median error, (**C**) median absolute error, and (**D**) trend time results. Statistical significance was determined by a Kruskal–Wallis test and a post hoc Dunn’s multiple-comparison test to compare performance metrics for each filtering type; all comparisons were non-significant (*p* > 0.05).

CRM predictions from the WHD Triceps demonstrated improved tracking of CRM_GT_ compared to the clavicle location, particularly when appropriate filtering was applied (Figure 7A). The raw signal showed substantial deviation from the ground truth, while all the filtered signals more closely tracked the expected CRM decline during progressive LBNP steps.

MdE analysis showed negative bias across all filter conditions (Figure 7B). The raw signal showed the least bias at −15.95%, followed by Custom (−16.78%), Chebyshev Type II (−22.44%), and Butterworth (−23.00%). MdAE ranged from 31.17% to 36.23% (Figure 7C), with Butterworth filtering achieving the best performance (31.17%), followed by Chebyshev Type II (32.35%), Custom (33.48%), and raw (36.23%). Statistical analysis demonstrated that the MdE showed no statistical significance between all filtering conditions (All *p* > 0.9999). MdAE was also not significant for all filtering conditions compared to the raw signal (All *p* > 0.9999).

Trend detection time analysis showed that the WHD triceps sensor achieved partial success in detecting threshold crossings (Figure 7D). The raw signal showed the earliest trend detection times with a mean of 11.50 min, followed by Chebyshev Type II (7.21 min), the Butterworth (6.01 min) and, finally, the Custom filter (3.38 min). There was no significant difference between the raw signal detection times and the Butterworth (*p* > 0.9999), Chebyshev Type II (*p* > 0.9999), and the Custom filter (*p* = 0.3985).

#### 3.5.3. Clinical PulseOx

**Figure 8:**
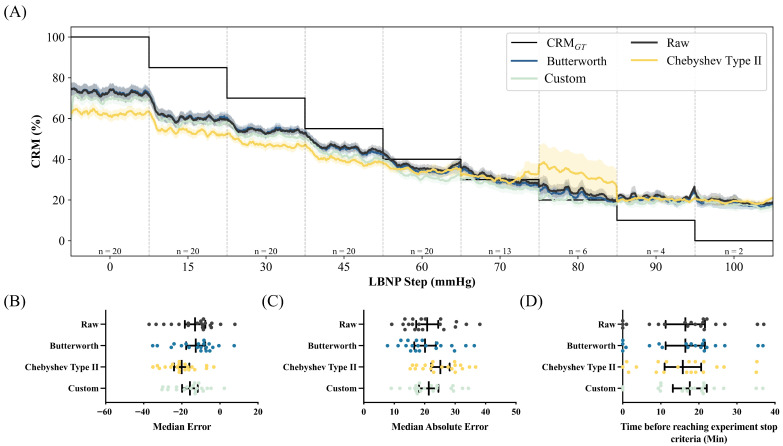
Results for Clinical PulseOx sensor-based CRM predictions. (**A**) Clinical PulseOx CRM over normalized LBNP steps across various filters compared against CRM_GT_. Performance for (**B**) median error, (**C**) median absolute error, and (**D**) trend time results. Statistical significance was determined by a Kruskal–Wallis test and a post hoc Dunn’s multiple-comparison test to compare performance metrics for each filtering type; all comparisons were non-significant (*p* > 0.05).

CRM derived from the Clinical PulseOx served as the reference and demonstrated strong agreement with CRM_GT_ across the raw signal and all filter conditions (Figure 8A). All signal outputs tracked the expected CRM decline during progressive LBNP. MdE analysis showed the lowest systematic bias among all sensor locations (Figure 8B). The raw signal showed a bias of −9.46%, closely followed by Butterworth (−9.95%) and Custom (−13.62%), while Chebyshev Type II showed the largest bias at −21.32%. MdAE ranged from 18.08% to 24.30% (Figure 8C), with Butterworth filtering achieving the best performance (18.08%), followed by raw (18.54%), Custom (19.31%), and Chebyshev Type II (24.30%). Statistical analysis demonstrated that the MdE showed no statistical significance between all filtering conditions (All *p* > 0.9999). MdAE was also not significant for all filtering conditions compared to the raw signal (All *p* > 0.9999).

Trend detection time analysis demonstrated reliable threshold detection across all filter conditions (Figure 8D). The Custom filter had the earliest trend detection time with a mean of 17.64 min, followed by both the raw and Butterworth, with both achieving an early detection time of 16.44 min, and finally followed by the Chebyshev Type II filter with an early detection time of 15.79 min. There was no significance between raw signal detection times and any of the filtering conditions (*p* > 0.9999).

### 3.6. Performance Comparison

To identify optimal configuration for each sensor location for comparisons, filter selection was based on MdAE. Based on this criterion, the raw signal was selected for the Clavicle location (bias = 38.08%), while Butterworth filtering was selected for both the Triceps location and the Clinical PulseOx (bias = 31.17% and 18.08%, respectively).

**Figure 9:**
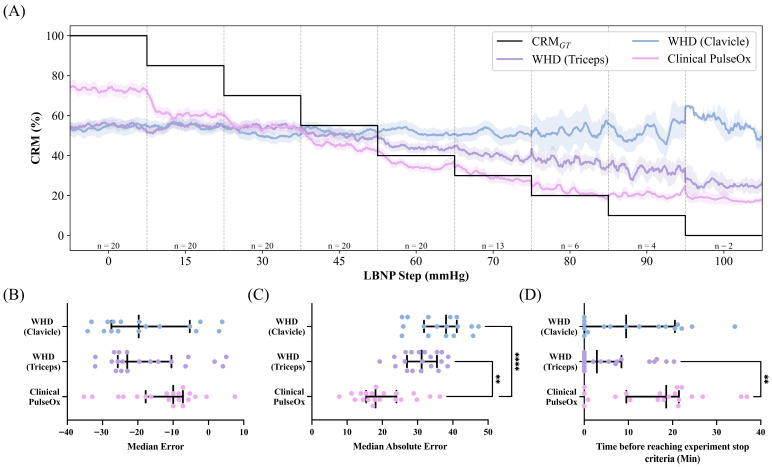
(**A**) Time series, (**B**) median error, (**C**) median absolute error, and (**D**) trend time of the raw clavicle WHD, Butterworth-filtered triceps WHD, and Butterworth-filtered Clinical PulseOx. Statistical significance was determined by a Kruskal–Wallis test and a post hoc Dunn’s multiple-comparison test to compare optimal sensor placement or type (** *p* ≤ 0.01; **** *p* ≤ 0.0001).

Figure 9 summarizes the performance comparisons across sensor locations using these optimal configurations and displays the time-series plot of all the optimal configurations for each sensor (Figure 9A). MdE demonstrated systematic underestimation across all sensors (Figure 9B), with Clinical PulseOx showing the least bias (−9.95%), followed by WHD Triceps (−15.95%) and WHD Clavicle (−19.74%). For MdE, statistical analysis showed no significant difference between any of the sites (WHD Clavicle vs. WHD Triceps: *p* > 0.9999, WHD Clavicle vs. Clinical PulseOx: *p* = 0.4133, WHD Triceps vs. Clinical PulseOx: *p* = 0.5104). MdAE revealed significant differences between sensor locations (Figure 9C). The Clinical PulseOx sensor achieved the lowest MdAE (18.08%), significantly outperforming WHD Triceps (36.23%, *p* < 0.0014) and Clavicle (38.08%, *p* < 0.0001) locations.

Trend detection time demonstrated differences between sensors in a potential clinical utility for CRM (Figure 9D). The Clinical PulseOx detected physiological deterioration with the greatest consistency across subjects, while the triceps WHD showed moderate detection capability. The clavicle WHD demonstrated limited utility, with most subjects showing minimal or no detection prior to reaching stop criteria. There was no significance between WHD Clavicle compared to WHD Triceps (*p* = 0.3299) or WHD Clavicle compared to the Clinical PulseOx (*p* = 0.3404). There was a significant difference between WHD Triceps and the Clinical PulseOx with a *p*-value of 0.0027.

## 4. Discussion

Hemorrhagic shock remains a leading cause of potentially preventable death in both civilian and military prehospital trauma [1,2,46]. Delayed intervention is associated with higher mortality outcomes [47], highlighting the need for early recognition and intervention. This requires continuous monitoring, which can be limited during mass casualty and prolonged field care scenarios. In addition to needing continuous monitoring, there is a need for objective monitoring technologies capable of detecting physiological signal changes associated with the onset of hemorrhagic shock [48]. CRM offers the potential for early detection of the onset of hemorrhagic shock. The CRM AI model utilizes CNNs to automatically extract features from physiological signal waveforms to detect early signs of hemodynamic decompensation. Wearable sensors are a strong substitute, since they provide constant monitoring without the need for bulky patient-monitoring systems [49]. While wearable technologies, such as the EPIC system, can allow for continuous, unobstructive physiological signal monitoring, the signal quality and compatibility with the CRM system was evaluated.

Signal-quality analysis demonstrated differences between sensor placements and filtering approaches. SNR showed no significant differences between the two site locations compared to the Masimo clinical PPG sensor, indicating that, on average, the signal had no issues with noise artifacts overpowering the signal being recorded. Although median SNR values were comparable across sensor placements, the triceps distribution demonstrated a downward skew, suggesting a subset of subjects with substantially lower SNR values and likely reflecting individual differences in tissue thicknesses based on measurement site [50], vascular density [51], and sensor coupling across the subjects [52]. For SQI, the triceps placement achieved values comparable to the Masimo reference when a Chebyshev Type II filter was applied, indicating that appropriate signal processing can overcome potential limitations of non-traditional PPG measurement sites. In contrast, the clavicle placement site demonstrated significantly lower SQI across all filtering conditions, likely attributable to anatomical factors of placing the sensor in the clavicle region, such as reduced superficial capillary density relative to peripheral sites [53]. Consistent with this, raw signals exhibited the lowest SQI across all three sensor placements, as baseline wander and DC offset in unfiltered waveforms introduce beat-to-beat shape variability that reduces template correlation independent of sensor location. The filtering performed isolates the AC pulsatile component, improving morphological uniformity and yielding a higher SQI score, which was observed across all sensors regardless of placement. The high SQI variability with the Custom filter at the triceps site suggests inconsistent performance across subjects, warranting further optimization of device-specific filtering algorithms.

The heart-rate correlation analysis corroborated the results of the signal-quality analysis. The triceps placement proved to be a viable location for the EPIC WHD, achieving a maximum coefficient of determination of 0.63. While this indicates a moderate correlation, it notably underperformed compared to the commercial Clinical PulseOx reference, which consistently maintained strong agreement regardless of the filter applied. In contrast, the clavicle placement demonstrated negligible correlation across all processing methods, effectively ruling it out as a reliable placement for monitoring with the current EPIC sensor configuration. These findings align with previous studies demonstrating that PPG heart-rate calculations vary substantially across body locations due to differences in vascular density, tissue composition, and susceptibility to motion artifacts [54,55].

The CRM model performance revealed that the Clinical PulseOx maintained robust CRM estimation across all filtering conditions, consistent with its role as a clinical-grade device optimized for PPG acquisition. The triceps EPIC WHD achieved partial CRM tracking when paired with appropriate signal processing, with Butterworth filtering providing the best balance between accuracy and trend detection capabilities. Notably, the filters that optimized heart-rate extraction (Chebyshev Type II) did not optimize CRM performance, suggesting that different waveform characteristics are relevant for this distinct application. This contrast may stem from the fact that the CRM CNN architecture was trained on high-fidelity waveforms with consistent morphology [56]. Wearable PPG acquisition introduces signal variability not present in those training conditions, as motion artifacts, tissue optical properties, and sensor-skin coupling collectively alter waveform morphology in ways that may challenge CNN feature extraction and affect predictions. In this context, the sharper roll-off characteristics of Chebyshev Type II filtering inadvertently distort the subtle waveform features that the model relies upon for compensation estimation [57]. The EPIC WHD placed at the clavicle failed to generate reliable CRM outputs even with various filtering conditions. Importantly, despite reduced absolute accuracy compared with the Clinical PulseOx, the triceps EPIC WHD demonstrated the ability to capture directional trends in CRM during progressive hypovolemia. From a clinical perspective, early trend detection may be sufficient without absolute CRM accuracy, as a clinician may just require an indication that physiological reserve is declining.

The promise of the triceps-placed wearable PPG compared to the finger-based Clinical PulseOx has important implications for prehospital hemorrhage monitoring. Finger-based pulse oximetry, while accurate, presents practical limitations in operational environments. Pulse oximetry sensors are susceptible to movement and dislodgement artifacts in prehospital environments [58,59], finger perfusion decreases during peripheral vasoconstriction associated with hemorrhage [60], and finger pulse oximetry may be compromised by injury or abnormal health conditions [61,62]. A sensor worn on the triceps offers potential advantages, including secure placement, protection from environmental interference, and continuous monitoring capability without impeding or interfering with manual tasks or patient care activities.

There are additional limitations worth discussing. First, LBNP induces central hypovolemia through blood redistribution rather than actual blood loss, which may result in physiological differences [63]. However, LBNP has been shown to mimic metabolic, immune, respiratory, autonomic nervous system, endocrine, and coagulation responses compared to actual hemorrhage, so this limitation may be minimal [64]. Second, our study population consisted of healthy volunteers in a controlled laboratory environment. From an initial testing evaluation perspective, healthy volunteers experiencing a controller-simulated hemorrhage allow for a much more consistent population for comparing WHD performance, but variability is much lower than in real-world situations. Future work will be required to expand WHD testing into real-world environments. Third, the pre-trained CNN model used for CRM estimation was originally developed on volume-clamp-derived arterial blood pressure waveforms before being extended to clinical-grade pulse oximeter PPG signals [42]. Wearable PPG signals acquired from non-traditional anatomical locations may not fully preserve morphological features that may have been present in the original training dataset. The decreased CRM performance observed in the raw wearable signals is consistent with this morphological mismatch, while improvement with filtered signals suggests that appropriate signal processing can partially recover physiologically relevant pulsatile features. Future work should evaluate CNN models trained directly on wearable PPG data from non-traditional anatomical locations to overcome this limitation. Finally, three subjects lacked clavicle sensor data due to technical issues, which introduces the potential for selection bias in comparing the two sites.

Due to the significant difference in performance between the two wearable EPIC WHDs, it is apparent that placement location plays a key role in signal quality and AI algorithm performance. While the triceps location performed well, other locations can also prove to be viable for clinical purposes to best leverage the ability of the reflective PPG to be placed anywhere on the human body. Further identification of locations that can provide strong signal quality is required to best take advantage of the EPIC WHDs. Future work should explore additional anatomical sites, including the neck region, where PPG-based hemodynamic monitoring has shown promise [65], to further characterize the EPIC WHD’s versatility across placement locations. Accordingly, additional testing of the WHDs across diverse clinical environments is also essential to confirm their accuracy and robustness in additional trauma-relevant scenarios.

## 5. Conclusions

This study carried out a proof-of-concept evaluation of the EPIC WHD for CRM-based hemodynamic monitoring during simulated hemorrhage. The triceps placement demonstrated viable performance for both heart-rate extraction and CRM estimation, while the clavicle placement proved unsuitable across all metrics. These findings support the potential feasibility of soft, flexible wearable sensors for continuous hemorrhage monitoring in operational environments where traditional finger-based pulse oximetry may be impractical. With further optimization of sensor hardware, signal processing, and an AI model specifically trained in wearable PPG signals, this approach may demonstrate a practical solution for continuous hemodynamic monitoring in prehospital and austere trauma environments.

## Figures and Tables

**Table 1:** Participant demographics and baseline values. Data presented as mean ± SD, *n* (%), or median (IQR).

Characteristic	Value
Participants, *n*	20
Age, years	37.40 ± 13.00
Sex, M/F	11 (55%)/9 (45%)
*Race*	
White	16 (80%)
Non-white	4 (20%)
*Ethnicity*	
Hispanic	4 (20%)
Non-Hispanic	14 (70%)
Not reported	2 (10%)
*Additional Info*	
BMI, kg/m^2^	26.10 ± 5.20
Resting SBP, mmHg	117 ± 16
Resting DBP, mmHg	77 ± 11
Resting HR, bpm	71 ± 11
Final LBNP Step Reached, mmHg	70 (60, 80)

## Data Availability

The data presented in this study are not publicly available because they have been collected and maintained in a government-controlled database located at the U.S. Army Institute of Surgical Research. These data can be made available through the development of a Cooperative Research and Development Agreement with the corresponding author.

## Supplementary Materials

The following supporting information can be downloaded at: https://www.mdpi.com/article/10.3390/s26082513/s1, Figure S1. Representative 5-second window of a Raw PPG waveform recorded from the WHD Clavicle (Top), WHD Triceps (Middle), and Clinical PulseOx (Bottom).

## Abbreviations

The following abbreviations are used in this manuscript:

CRM	Compensatory Reserve Measurement
EPIC	Epidermal Patch for Imperceptible Care
LBNP	Lower-Body Negative Pressure
AI	Artificial Intelligence
CNN	Convolutional Neural Network
WHD	Wearable Healthcare Device
PPG	Photoplethysmography
IMU	Inertial Measurement Unit
IRB	Institutional Review Board
ECG	Electrocardiogram
FHE	Flexible Hybrid Electronics
LED	Light-Emitting Diode
RMS	Root Mean Square
SNR	Signal-to-Noise Ratio
SQI	Signal Quality Index
IIR	Infinite Impulse Response
FIR	Finite Impulse Response
R^2^	Coefficient of Determination
MdE	median error
MdAE	median absolute error
ANSI	American National Standards Institute
AAMI	Association for the Advancement of Medical Instrumentation
SD	Standard Deviation
IQR	Interquartile Range
BMI	Body Mass Index
SBP	Systolic Blood Pressure
DBP	Diastolic Blood Pressure
HR	Heart Rate
