# Supplementary material for: Validation of a Wearable Photoplethysmography-Based Sensor for Compensatory Reserve Measurement Monitoring in Simulated Human Hemorrhage
Source: Sensors (Basel). 2026 Apr 18;26(8):2513. doi: 10.3390/s26082513 (PMC13120294; doi:10.3390/s26082513)
Supplement: Supplementary file 1 [file sensors-26-02513-s001.zip › sensors-4231145-supplementary.pdf]

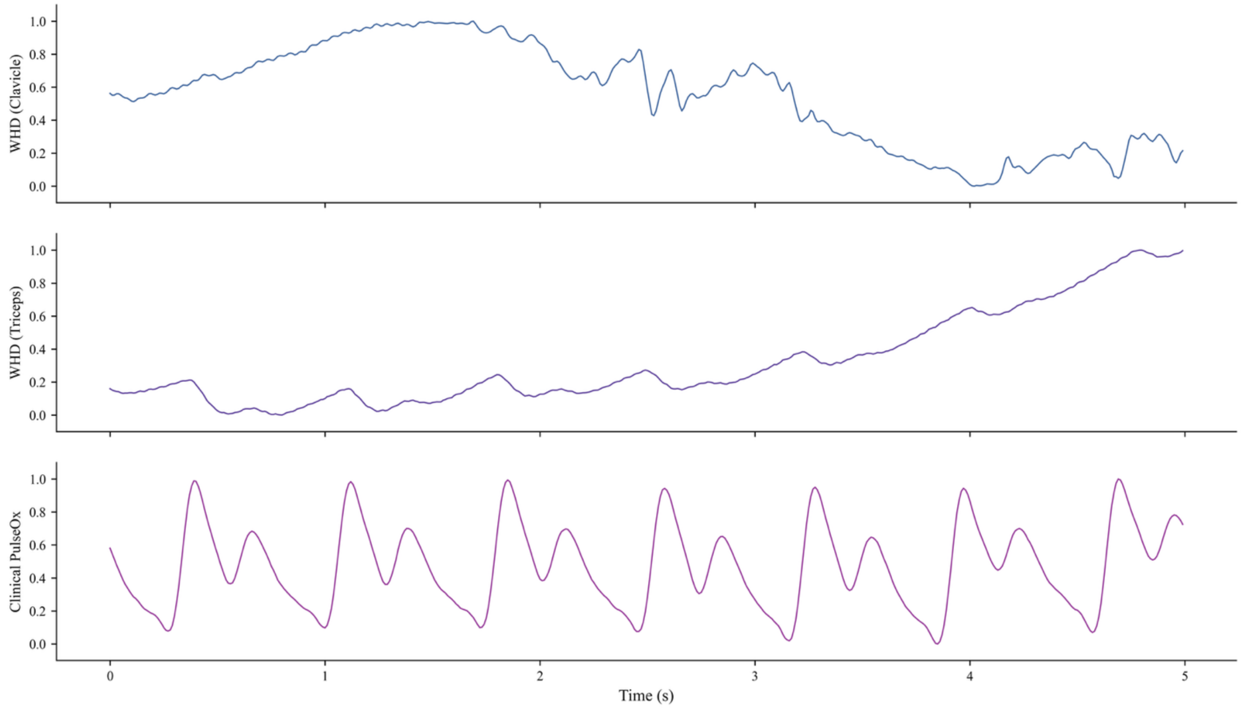

Figure S1. Representative 5-second window of a Raw PPG waveform recorded from the WHD Clavicle (Top), WHD Triceps (Middle), and Clinical PulseOx (Bottom).
